# Why do we bother? Exploring biologists' motivations to share the details of their teaching practice

**DOI:** 10.12688/f1000research.6129.1

**Published:** 2015-02-17

**Authors:** Graham Scott

**Affiliations:** 1 School of Biological, Biomedical and Environmental Sciences, University of Hull, Hull, HU6 7RX, UK

**Keywords:** teaching, practice, biologist, scholarship of teaching and learning

## Abstract

There exists in the UK (and across the global HE sector) a community of practitioners who define themselves as biologists but who are more than that. They are reflective educators involving themselves in the Scholarship of Teaching and Learning (SoTL). In this paper I explore the motivations of these individuals to disseminate the detail of their teaching practice. I reflect upon my own experience and my observations of the experiences of others and in doing so I explore common enablers/disablers to engagement with SoTL. I discuss the prime importance of a supportive disciplinary SoTL community and of inspirational individuals (peers and managers alike). I reflect upon the tensions that exist between teaching and research focused career paths and I consider the possibility that this tension is of variable significance. I conclude that the barriers to individual engagement with SoTL can be overcome and that the individual drive to do so is a powerful one.

## Introduction

During one of the early planning sessions for the Society of Experimental Biology Symposium *Teaching and Communicating Science in the Digital Age *(15 ^th^–17 ^th^ December 2014, Charles Darwin House, London, United Kingdom) it occurred to me that as we discussed what digital communication might mean, how it might be utilised to increase our personal profiles as practicing biologists and how it might be used to change the ways in which we disseminate our science and teach our students, we seemed to routinely miss one area of our day to day activities namely the dissemination of teaching practice itself. Not what we teach, but rather how we teach and how we evaluate and disseminate our teaching practice.

Here I am not primarily interested in the *mechanisms* by which we disseminate our teaching practice – we illustrate symposium presentations using digital media, we disseminate through social media, we blog and we submit papers to online journals for example. Instead my primary focus is a desire to better understand our *motivations* to share our practice. In so doing I hope to gain some insight into our experiences as professionals who are often be viewed as being at the intersection of two areas of academic practice, teaching and research, and at the boundary that exists between disciplinary areas (in this case the biosciences, the social sciences and the educational sciences).

Through discussion and reflection it became apparent to me that the motivation to disseminate ones teaching practice is bound up with the development of an individuals scholarship, the key enablers/disablers we each experience, and the audience to whom one “speaks”. These three areas are therefore the secondary foci of this paper.

This is in large part a personal reflection, an attempt that I have made to make sense of my own motivations and the professional pathway that I have undertaken and continue to follow. I acknowledge therefore that this is a very UK-centric viewpoint and that the breadth of my experiences limits it. I have tried to reflect upon my practice in the context of the following:

1. My reflections upon my own pedagogical practice and dissemination activities and my observations of those of my peers.
2. My discussions with colleagues who are new to teaching (as a mentor to newly appointed lecturers in my own institution over more than 10 years; and, as a facilitator of several new to teaching CPD events organised by both the Higher Education Academy and the Society of Biology).
3. My discussions with the authors of papers about the teaching of biology in my role as an associate editor and editor of journals.
4. My readings of the relevant literature (and in particular for example the work of Healey, 2000; Lave & Wenger, 1991; Trigwell *et al.*, 2000; Wenger, 1998).
5. Interviews with seven academic colleagues based in the UK (see below).

## The interviews

I carried out seven interviews with UK-based Higher Education (HE) bioscience educators to provide myself with a sounding board against which to test my own reflections. The interviews were conducted by telephone and interviewees were selected because I was aware, or I was made aware, that they actively engaged in the dissemination of their teaching practice. All but one of the interviewees were, or had until recently been, employed on traditional teaching and research (T&R) contracts. Of these, three I considered to be established (two Chairs – both awarded on the basis of teaching/engagement rather than biological research, and a Senior Lecturer; one female, two male), and three I considered to be new to teaching (having less than four years formal experience) (one female and two male). The final (female) interviewee had not held a T&R contract but had more than 15 years of formal teaching practice in mainstream UK HE Biology departments and a wealth of experience in both the Scholarship of Teaching and Learning (SoTL) and the professional development of teaching faculty within the biosciences. The interviewees represented mainstream bioscience departments at UK HEIs from the Russell Group, the post 1992 group and other institutions from three of the four UK home nations and so were drawn from across the sector. (I accept however that they could in no way be described as a fully representative sample). The relevant ethics committee of the University of Hull approved this data collection.

Interviews were conducted by telephone and lasted between 30 and 40 minutes each. The discussion was recorded and transcribed and interviewees were provided with an opportunity to comment upon their own transcript. The discussion was semi-structured focusing upon four broad areas each phrased as questions that were sent to the interviewees in advance:

1. Can you describe to me the ways in which you have communicated your teaching practice to colleagues. How has this changed over time?
2. Who do you consider to be the audience when you disseminate your teaching practice?*
3. At what stage in your career did you first feel the need to tell colleagues formally about your teaching practice? What motivated you to do so?
4. Have there been personal costs/benefits associated with the efforts that you have gone to share your teaching practice with others? To what extent have you been encouraged/discouraged in your efforts (and by whom)?

* This question was not sent to interviewees in advance. It arose during the first interview and was subsequently added to the other discussions at an appropriate juncture.

These questions were asked/discussed in the context of a wider discussion about SoTL and about the career trajectory of the individual concerned.

## Disseminating our teaching practice

The dissemination or communication of ones teaching practice is a key dimension of Trigwell *et al.* (2000) four dimension model of the Scholarship of Teaching and Learning ( Table 1). As the model illustrates some teaching faculty (those at level 1), who see teaching in a teacher-centric way, do not communicate their practice and demonstrate limited awareness of the scholarship of others. On the other hand, some members of the faculty move far beyond this and see teaching as a student-focused activity. These colleagues publish the outcomes of their scholarship in the international peer-reviewed literature and in doing so demonstrate a capacity to conduct action research and an advanced knowledge of the pedagogic literature. The transition from level 1 to level 4 is not a pre-requisite of teaching, nor is it a necessary consequence of teaching activity. Rather it represents the potential ontogenetic pathway of a reflective practitioner as they negotiate *Threshold Concepts* (see Meyer & Land, 2003) and overcome the barriers imposed by *Troublesome Knowledge* (A. Tierney, *pers. com*.).

**Table 1.** Multi-dimensional model of scholarship of teaching (adapted from Trigwell *et al.*, 2000).

Level	Informed	Reflection	Communication	Conception
**1**	Uses informal theories of teaching and learning	Little reflection or unfocused reflection	Does not communicate practice	Believes teaching to be teacher-focused
**2**	Engages with the general literature of teaching and learning		Shares practice informally or in a local context (tearoom conversations; departmental seminars)	
**3**	Engages with the disciplinary pedagogic literature	Demonstrates reflection-in-action	Presents practice at local/ national conferences	
**4**	Conducts action research, has synoptic capacity and pedagogic content knowledge	Reflection focused on asking what do I need to know about x here, and how will I find out about it?	Publishes in international scholarly journals	Believes teaching to be student-focused

Over recent years, I have observed the practice of many of my peers as it has developed from level 2 through level 3 and into level 4 of the Trigwell *et al.* model; a journey that I have shared with them. Three of my four more established interviewees described their dissemination activities and the materials presented as having evolved over time. Their initial disseminations were spoken rather than written papers and they tended to be quite descriptive. Their more recent work has been more likely to be underpinned by pedagogic theory and robust data, but interestingly none of them saw themselves as having made a transition from being biologists to researchers in education. One down-played their grasp and use of the literature to inform their work (comments that I took to indicate a level 2 Informed dimension and a level 3 Communication dimension from Table 1). Another explained that they still felt less comfortable with an audience of what they described as social scientists, by which I understood them to mean non-biologists interested in SoTL in comparison to an audience of biologists with an interest in SoTL. This was because they felt that they lacked the grounding in SoTL that a PhD and Post-Doctoral Research Associates (PDRA) track had provided for them in biology. This is a common perception and one that has been discussed by Kelly *et al.* (2012), who suggest that this is an issue that goes beyond disciplinary research methodology differences and is intimately bound up with the disciplinary identities of the individuals involved. My interviews with new to teaching faculty provide an indication that a subtle shift might be taking place as a result of changes in the way in which UK HEIs develop new academics. Although two of them described spoken presentations in which they explained to colleagues what they had done and suggested that this was a sharing of good practice rather than a presentation of research (level 2 communication dimension in Table 1), the third (on a standard T&R contract) described the fact that as part of a compulsory Postgraduate Certificate (PGCert) in HE practice they were *required* to undertake a piece of action research involving the re-development of a module. As a minimum this project had to be disseminated as a presentation to their peer group, but in this case it resulted in a publication in the journal *Bioscience Education*. As a journal editor handling papers on biology education I believe that this is becoming more commonplace. It is possible therefore that an emphasis upon SoTL from the outset as part of the routine practice of an academic may provide an opportunity for some individuals to enter the Trigwell model at a higher level and perhaps therefore subsequently progress at an accelerated rate (this is a possibility that I will return to later in this paper).

## The audience

From the preceding section it is clear that the primary audience for the dissemination of SoTL is one’s peers. Commonly we start by discussing our ideas with a mentor or other key colleague (see below) and from there move on to present posters and oral presentations at events organised within our institution. Our audience grows as we develop as SoTL practitioners and we begin to present our work at national and international meetings, through publications and via social media. But it would be wrong to think that this has always been an easy journey to make. Consider the following quotation taken from an interview with an established academic:

“ *Being keen on learning and teaching was, certainly when I started out, it was almost you know, it was something you kept under your hat.*” Established interviewee.

This statement typifies an experience common to many of us who started on our academic careers 15–20 years ago. At that time (in a UK context) opportunities to formally present our teaching practice were significantly less common than they are today, particularly in the pre-digital world when outlets such as blogs and social media were not available to us. Two of the more established interviewees discussed the critical importance of their disciplinary learned societies in this regard, both having had the opportunity to present their pedagogy in education themed sessions at disciplinary research conferences. Both talked about the importance of these sessions as places to both present ones own work but also to hear about the good practice of others. These meetings were seen as a way to avoid the redundancy of “ *reinventing the wheel*”. On the face of it this is a positive state of affairs but the same interviewees also recollected that at that time (15 to 20 years ago) these sessions were seen as “ *a bit of a side show to the main* [disciplinary research] *event*”. Common to the experience of all of my interviewees was the perceived importance of an audience of peers beyond their own institution (be that the readership of a journal or blog, the users of social media, or the participants of a symposium or conference). All of them described the importance of being part of a supportive community. One explained:

“ *One* [benefit of making the effort to disseminate ones teaching practice] *is becoming involved with the really good community of like-minded people who are very supportive and good to be with and good to discuss with. You’ll always have a good debate but it’s very collegial and I think one of the real differences that I’ve noticed between presenting at research conferences, certainly in my discipline, and learning and teaching ones is that collegiality. People aren’t trying to catch you out and prove how clever they are. They’re genuinely interested in what you have to say*.” Established interviewee.

However, all of the established interviewees expressed some concern that in the UK context at least, the perceived shift in focus of the Higher Education Academy away from disciplinary workshops and conferences towards more generic ones may stifle debate within the biosciences and limit opportunities for dissemination. There was even a suggestion that a golden age of UK SoTL may have passed. This is not a view that I necessarily share. Whilst I agree that the HEA Bioscience Subject Centre and the HEA STEM group were key to the development of a broader Bioscience SoTL community in the UK it is evident that that community did exist in nascent form prior to that period and that through its own efforts can continue to thrive. We have seen that the learned societies have played (and continue to play) a role in this by providing opportunities for SoTL and the dissemination of SoTL alongside disciplinary research. As long as SoTL does not remain/become a “ *side-show*” at their meetings there is a potential for continued activity. As one of my interviewees explained, it is essential that as a community of practitioners we do our part to maintain the development of a culture in which SoTL is seen as being on a par with research across the HE sector. This is perhaps particularly important given that it was recently estimated that as many as 25% of UK academics are described as “teaching-only” (Times Higher Education, 2008) many of whom are contractually obliged to engage in “scholarship” ( Cashmore, 2009).

One outcome of my interviews, although not a surprise, was something of a disappointment. With the exception of the established interviewee who is currently primarily involved in the teaching of pedagogy and SoTL to in-post academics and one who was new to teaching, none of the interviewees mentioned their students as an audience for their SoTL outputs. When pressed all explained that they regularly told their students how learning would take place (i.e. they described the various learning strategies employed) and that their own students were often involved (as participants) in their SoTL activity, but only the two of them reported using the outcomes of SoTL activity (their own or that of others) to demonstrate to their students the benefits of the pedagogy that they employed. My wider experience of discussing this with colleagues suggests that not including ones students as part of the SoTL audience is the norm rather than the exception. However, my personal experience is that doing so can be a useful exercise. It helps one to reflect upon the pedagogies employed and upon ones own scholarship. It can also provide students with a useful insight into their own learning experience ( *pers obs*).

## Motivations

So what makes a biologist want to talk about how to teach biology rather than about biology *per se*? By this I don’t mean why do we have the coffee room or water cooler type discussions that are commonplace in any educational setting about how a session has gone or might be improved, or how a course could be changed to take into account a development in the field or a new administrative requirement. I mean why do a minority of biology faculty (and in most institutions it is a minority) take that extra step and start to formally share their practice in the same way that they might be expected to share the details of their disciplinary research.

For many of us (myself included) an inspirational colleague or mentor set us on our path. In some cases they modelled good practice themselves; they were innovators who took the time to explain to us why they were changing the way they taught. Their passion for innovation and willingness to share the details of both their success and their failures provided us with a model to follow. In other cases supportive mentors and senior colleagues provided us with a space in which to work – perhaps they gave us free rein to attempt a new way of teaching or perhaps they encouraged us to undertake CPD that exposed us to the possibilities of teaching in a different way. These key motivational figures were common to both the established and new to teaching interviewees.

All of the interviewees and many of the new to teaching academics that I have discussed this with share a common desire to improve their own teaching and to enhance the student experience. But whilst this clearly underpins the reflective development of teaching and the motivation to share good practice, it is not in itself a sufficient explanation of the drive to disseminate – after all, it would be perfectly possible for a lecturer to strive to do both without ever presenting their practice to colleagues. Several interviewees described the presentation of their own practice almost as an obligation, a way of making a contribution to the community from which they themselves benefit:

“ *I wanted to keep attending those sort of events … and it seemed only fair to also contribute if you’re going to listen. It’s pretty rare that I would attend any event without offering to speak at it.*” New to teaching interviewee.

There was also a sense that people wanted to present a realistic account of what worked or did not work. To do more than showcase their outputs by giving an honest “warts and all” account of the difficulties encountered. This once again emphasises the importance of a feeling of collegiality and belonging to an active community. Being part of the group is in itself a clear motivation for many to continue to disseminate their work.

All of the interviewees gave a very positive account of their role within an established or growing community of scholars and demonstrated a strong commitment to SoTL at whatever level of engagement they had achieved. I have already identified the importance of the community to established biology faculty with a history of SoTL type activity but I suspect that it is also very important to the new generation of academics in the UK for who SoTL is a requirement of a Teaching and Scholarship type contract. None of the established interviewees thought that it would have been possible to be awarded a Chair on the basis of Teaching and Scholarship in their own institution until relatively recently and all saw the formal recognition of excellence in teaching that has become more prevalent in the UK HE sector in recent years as a positive development. Clearly then career development (promotion) has become a new motivation for engagement with SoTL and the dissemination of teaching practice in recent times – particularly in the case of the 25% of UK academics are described as “teaching-only” (Times Higher Education, 2008) and those on teaching and scholarship contracts who are contractually obliged to engage in “scholarship” ( Cashmore, 2009) previously mentioned. These colleagues have the potential to become a mainstream SoTL community within the biology discipline and are likely to continue to be motivated to undertake SoTL activity throughout their careers.

However, this may not be a sufficient motivation for a continued involvement in SoTL for those colleagues who remain on traditional teaching and research contracts. As I have previously mentioned, I have recently observed the development of a distinct sub-community of academics who discuss the preparation of a presentation or a paper related to their teaching practice as a key hurdle to be jumped on the road to promotion via a teaching and research route. This is particularly the case amongst many new to teaching academics on T&R contracts who have undertaken what could be described as a SoTL activity as part of a PGCert (or similar qualification/training course). It is possible, as I suggested previously, that these colleagues are motivated to engage in a meaningful way with SoTL and effectively enter the Trigwell *et al.* (2000) model at a higher level. But it may also be possible that as a result of their potentially narrow range of motivation to disseminate they do not fully establish themselves across the breadth of the dimensions of the model.

## Enablers/Disablers

For those employed on a traditional teaching and research contract the tensions existing between research and teaching (and in particular the lack of time to do both) are a key barrier to SoTL. There is for these colleagues a significant pressure to win disciplinary grant funding, to write high impact research papers and to teach well. For many the extra efforts involved in the dissemination of teaching practice may simply be beyond them. Some rare individuals do manage to maintain both a disciplinary research profile and become very active within the bioscience education community, but for many a choice is made along the way to specialise in one area or the other. For some the choice is less problematic than for others. Those on a teaching and research contract are contractually obliged to carry out research and it is very unlikely that SoTL activities would be seen as research in that context. This does not mean that the journey from biologist to scholar of biology teaching is an easy one for those who are contractually empowered to make it. Some of the colleagues I have discussed this with describe a sense of guilt that they are turning their backs on aspects of their disciplinary background. Rowland (2012) has expanded upon this problem in her discussion of the fact that some academics do not see those who specialise in teaching rather than in research as being sufficiently current to deliver advanced classes and supervise students undertaking disciplinary research projects. Others explain that they feel like imposters in a new field. Self-doubt may therefore be a key barrier that prevents some colleagues from achieving their full potential.

As has been previously mentioned, particular people are often a key enabling factor in the development and maintenance of an individuals motivation to engage in SoTL and to disseminate ones teaching practice. I have already indicated the importance of inspiring mentorship and of participation in a supportive community of practice. However, other inter-personal relationships are important too. Several interviewees discussed the significance of strong leadership at the institutional level. One established interviewee commented that it “ *took a brave Vice Chancellor to establish those routes*” (teaching and scholarship/engagement promotion pathways to the Professorial level) and added that it then took “ *people who really wanted to develop their careers in teaching to actually then take it forward*”. A new to teaching interviewee described how their Vice Chancellor had instigated a series of teaching awards that were celebrated alongside the achievements of graduating students. This very public recognition of teaching excellence was described as bringing value to the efforts that academics make. However, whilst reward and recognition in the form of awards was seen as a general positive and as a validation of the value of individual practice, the view was also expressed that institutional notoriety could be short-lived. One established interviewee, themselves the recipient of numerous institutional and international awards to recognise excellence in teaching, expressed the view that in their personal experience there remained a disconnect between this type of recognition and rewards linked to institutional promotion criteria. Middle-managers and line-managers were also described as being very important enablers and/or disablers. Several interviewees (and many of the colleagues I meet through my facilitation of workshops) describe the support that a good line manager provides as being essential to their engagement with SoTL and key to their ability to disseminate their practice. Often this support is very practical, taking the form, for example, of the financial support needed to undertake SoTL activities and to attend dissemination events. Sometimes it is simply being given the time and space in which to develop this facet of an individuals practice. On the other-hand an unsupportive line-manger is often cited as being a complete barrier to SoTL. In the case of reward of SoTL through promotions processes one established interviewee expressed the view that although new criteria had been established to recognise excellence in teaching they were likely to be used by panels of academics who had themselves been promoted on the basis of research and may not understand how to apply them. Whilst such a situation would undoubtedly be problematic robust promotional systems and criteria should mitigate against this.

## Conclusion

I titled this paper “ *Why do we bother*?” a question that at the time I thought was a neutrally phrased one. Early in the writing of the paper however I was challenged by an interviewee who assumed that I had intended it to be read in a negative fashion. As a result I have realised that in fact my preconception was that my reflections on the topic would reveal to me a very simple picture – we bother because we enjoy it, because the work we do has value, and quite simply because we can’t imagine not doing it. I think that on the whole I have found evidence to support my view but I have also revealed to myself that the picture is not cut and dried.

There exists in the UK (and across the HE sector) a community of practitioners who define themselves as biologists but who are more than that. We are reflective educators involving ourselves in the Scholarship of Learning and Teaching. Ours is a self-sustaining community and one that at the moment is enjoying the benefit of a period of supported growth (recently primarily supported in the UK context by the HEA Bioscience Subject Centre). It is in a position to continue to grow as students and student driven market forces apply pressure upon institutions but we should not be complacent. I would add to all of the motivations that have been discussed above the part that disseminating our practice can play in show-casing our work and our worth to the senior managers and policy makers who have the future of our community partly in their hands.

As a closing thought, lack of support has been seen as a key barrier to individual engagement with SoTL, but lack of support can be overcome if the motivation to do so is sufficient. As one established interviewee commented, “ *somebody said to me what would happen if your line manager said you couldn’t do this* [SoTL] *anymore? And I said, well I’d do it anyway.*”
