# Supplementary material for: Why do we bother? Exploring biologists' motivations to share the details of their teaching practice
Source: F1000Res. 2015 Feb 17;4:46. [Version 1] doi: 10.12688/f1000research.6129.1 (PMC5765384; doi:10.12688/f1000research.6129.1)
Supplement: Peer review history

# Peer review: Referee response for version 1

**DOI:** 10.5256/f1000research.6567.r8753

**Published:** 2015-05-26

**Authors:** Vivien Rolfe

**Affiliations:** 1 University of the West of England, Bristol, UK

**Title and content:**

A paper of interest to the ‘bioscience’ community exploring why we share our teaching practices with others? This is very topical in light of changes to the HE sector and the decline in support and project funding for education research. So I am hoping the paper will enlighten me on not just the ‘why’ but ‘how do we bother’ to become excellent practitioners and share to support our subject communities?

**Methodology:**

A small number of interviews were conducted reflecting a range of institutions. It wasn’t clear how the author got from interview transcripts to aligning to Trigwell’s model of scholarship of teaching? How was the data analysed and was any particular software used? The findings are discussed in accordance with the interview lines of enquiry.

**Discussions and conclusions:**

I find it a little strange that none of the interviewees or reflections by the author mention ‘open education’ as a motivational factor and enabler for sharing teaching practices? The advent of open licenses and easy technologies has made it simple for people to share news and practices to global audiences and reach out to learners beyond their institution. I think the global open education movement is a significant driver and possibly worth a sentence in the discussion that it was not mentioned by any of the interviewees.

Under motivations also I’m surprised that seeking professional fellowships (e.g. HEA / SEDA UKPSF or HEA National Teaching Fellowships) are not mentioned as a driver, as teaching excellence is a National key performance indicator and many institutions have set staff targets for these. The need for teaching staff in all disciplines to reflect and enhance their practice has grown rapidly, so again, it might be worth a comment noting that this was not mentioned during the interviews.

A final area I’m surprised was not raised is the lack of funding for education projects in the UK. Changes in direction for organizations including the HEA and Jisc have had a major impact on financial support for teaching innovation and the ability to build communities around projects as used to take place.

Overall, a well conducted study addressing a very topical question that will be of interest to the biology and bioscience community.

I have read this submission. I believe that I have an appropriate level of expertise to confirm that it is of an acceptable scientific standard.

# Peer review: Referee response for version 1

**DOI:** 10.5256/f1000research.6567.r8674

**Published:** 2015-05-22

**Authors:** Catherine J. Hack

**Affiliations:** 1 School of Biomedical Sciences, University of Ulster, Coleraine, UK

This is an interesting and timely piece, given the current drivers to enhance teaching quality across the UK Higher Education sector, and the growth in discipline specific pedagogic research over the last decade.

This paper is based on a small qualitative study, which used semi-structure interviews with seven participants. Although a small cohort, it was purposefully recruited to include an appropriate range of experience, age and gender balance. The methodology was appropriate for the objective of the study which was to explore the motivations of academics, specifically those involved in research and teaching in the biosciences, to evaluate their teaching and share their practice. The limitations of the research were identified. The analysis was informed by Trigwell’s multi-dimensional model of scholarship of teaching and was situated within the authors’ extensive experience of publishing pedagogic research and scholarship in the life sciences. I would be interested to hear whether the author agrees with others (e.g. Stierer, 2007,and Tight, 2013) that there has been significant change in the quality of scholarship and pedagogic research conducted by discipline specialists (i.e. not educationalists) and whether discipline-specific researchers are conducting more robust studies which are informed by the relevant pedagogy- i.e. moving beyond Trigwell's model.

The author comments on his choice of title, and I wonder whether a change to ‘why we bother’ may be more reflective of the article content?

I have read this submission. I believe that I have an appropriate level of expertise to confirm that it is of an acceptable scientific standard.
